# Global ocean freshening, ocean mass increase and global mean sea level rise over 2005–2015

**DOI:** 10.1038/s41598-019-54239-2

**Published:** 2019-11-27

**Authors:** William Llovel, S. Purkey, B. Meyssignac, A. Blazquez, N. Kolodziejczyk, J. Bamber

**Affiliations:** 1 LEGOS, CNES/IRD/CNRS/UPS, Toulouse, France; 2 Scripps Institution of Oceanography, La Jolla, CA USA; 3 LOPS, University of Brest/IFREMER/IRD/CNRS, Brest, France; 4 University of Bristol, Bristol, UK

**Keywords:** Physical oceanography, Physical oceanography, Physical oceanography, Physical oceanography

## Abstract

Global mean sea level has experienced an unabated rise over the 20^th^ century. This observed rise is due to both ocean warming and increasing continental freshwater discharge. We estimate the net ocean mass contribution to sea level by assessing the global ocean salt budget based on the unprecedented amount of *in situ* data over 2005–2015. We obtain the ocean mass trends of 1.30 ± 1.13 mm · yr^−1^ (0–2000 m) and 1.55 ± 1.20 mm · yr^−1^ (full depth). These new ocean mass trends are smaller by 0.63–0.88 mm · yr^−1^ compared to the ocean mass trend estimated through the sea level budget approach. Our result provides an independent validation of Gravity Recovery And Climate Experiment (GRACE)-based ocean mass trend and, in addition, places an independent constraint on the combined Glacial Isostatic Adjustment – the Earth’s delayed viscoelastic response to the redistribution of mass that accompanied the last deglaciation- and geocenter variations needed to directly infer the ocean mass trend based on GRACE data.

## Introduction

The planet Earth is experiencing a global warming due to an energy imbalance between the incoming solar radiation and the outgoing long wave radiation at the top-of-the-atmosphere. Based on satellite and *in situ* measurements along with numerical models for the year 2000 onwards, the decadal global imbalance has been estimated to be 0.5–1 W · m^−2^ ^1–3^.

Global mean sea level rise is one of the most direct consequences of global warming. Over the 20^th^ century, tide gauge records indicate a linear increase of the global mean sea level, with rates ranging from low estimates of 1.1 ± 0.3 mm · yr^−1^ (1σ)^4^, to high estimates of 1.7 ± 0.2 mm · yr^−1^ (1.65σ^5^, the quoted trend errors denote the standard deviation at the 68% -1σ- and 90% -1.65σ- confidence interval, respectively). Based on satellite altimetry since 1993, global mean sea level rise presents a higher rate of 3.3 ± 0.4 mm · yr^−1^ (1.65σ)^6,7^, denoting an acceleration in this rise over the 20^th^ century.

Sea-level rise is caused by ocean warming (i.e. expansion of sea water, the so-called thermosteric sea level) and the imports of fresh water from continents (i.e. ice sheets mass loss, mountain glaciers melting and land water change). The freshwater discharge refers to the barystatic sea level change or the net ocean mass change. Because of the high accuracy of the complementary observing systems, we are now able to close the sea level budget within the uncertainties by combining satellite altimetry data, ocean mass change from Gravity Recovery and Climate Experiment (hereafter GRACE) and *in situ* measurements of temperature^8,9^.

The net ocean mass change inferred from GRACE over the oceans presents the highest uncertainties in the sea level budget. GRACE data are extremely sensitive to solid Earth movements (i.e., the mass redistribution), in particular, Glacial Isostatic Adjustment (GIA) due to the last deglaciation that began 21000 years ago and to the geocenter motion^10^. GIA accounts for ~40–50% of the net barystatic sea level trend over 2003–2016 (see^10^). Both processes are not accurately known and are responsible of most of the uncertainty (0.12 and 0.21 mm · yr^−1^ respectively; 1.65σ)^10^, in the long-term net barystatic sea level trend, leading to an uncertainty of 0.27 mm · yr^−1^ (1.65σ)^10^. Reducing uncertainty is necessary to assess measurement accuracy of the barystatic sea level change and better constrain the sea level budget.

The ocean mass change can be assessed by other approaches. One approach consists in estimating the net import of continental fresh water from ice mass loss from ice sheets (Greenland and Antarctica), mountain glaciers melting and the land water change. This mass budget approach has recently been reevaluated over January 2004–December 2015^7^ and leads to a positive trend of 2.13 ± 0.14 mm · yr^−1^ (see^7^). Note that the quoted error does not account for systematic biases that can affect GRACE data such as GIA or the geocenter variations. This value significantly differs from a recent reevaluation of the net ocean mass from continental ice melting being 1.63 mm · yr^−1^ over the same time period^11,12^ and based on an ensemble of GRACE solutions being 1.56 ± 0.27 mm · yr^−1^ over 2004–2015 (1.65σ)^10^. This disagreement raises new questions about the confidence of the barystatic trend estimate for the recent years.

Estimating the global ocean freshening offers an alternative approach for estimating the net ocean mass changes^13–15^. Ocean freshening has been investigated for the past decades at the surface of the oceans at global and regional scales^16–19^. The motivation is to understand the long-term salinity changes and the link on global and regional water cycles. Because of the lack of *in situ* data, long-term salinity change in subsurface remains largely unknown. We estimate in this study the global ocean salinity change with all available *in situ* data over 2005–2015.

The global ocean freshens due to both floating sea ice melting (comprising Arctic sea ice and Antarctic ice shelves) and the continental freshwater input. Floating sea ice change does not affect sea level because of Archimedes’ principle while continental freshwater input affect sea level by adding mass into the ocean. Thus, in order to estimate the net ocean mass from ocean salinity, a correction has to be applied accounting for any changes in sea ice volume (see the method section). To estimate the ocean mass change based on a global freshwater budget, one can also do this in terms of sea level change by calculating the salinity contribution only (i.e., the halosteric contribution). This allows for easy comparison with other sea level rise budgets. Previous studies attempted to develop this approach, but had to rely on sparse salinity measurements (based on the World Ocean Database)^20^, for the past decades.

Since the beginning of the 2000s with the launch of the international Argo program, we now have access to an unprecedented global sampling of salinity and temperature measurements for the upper 2000 meters of the oceans^21^. The coverage of Argo floats is nearly global since the beginning of 2005 providing us the opportunity to reassess the recent ocean freshening over 2005–2015. In addition, considerable improvements have been made in estimating the present-day sea-ice volume change for the Arctic (based on the Pan-Arctic Ice Ocean Modeling and Assimilation System -PIOMAS-^22^, and satellite observations)^23^, and the Antarctic ice shelf volume changes (based on satellite observations)^24^, for the past decade. We note that Antarctic sea ice, in contrast to the Arctic, has shown only a minor change in volume during our study period (e.g.^25^).

The goal of the study is twofold. First, we attempt to place a new constraint on the global ocean freshwater budget over the past decade and, second, we evaluate the consistency of the ocean mass trend changes inferred by different methods. Finally, the latter comparison will bring a new constraint on the corrections (GIA and geocenter variations) needed to directly estimate the trend of the global ocean mass change measured by GRACE over the oceans.

## Results

### The global mean sea level budget

**Figure 1:**
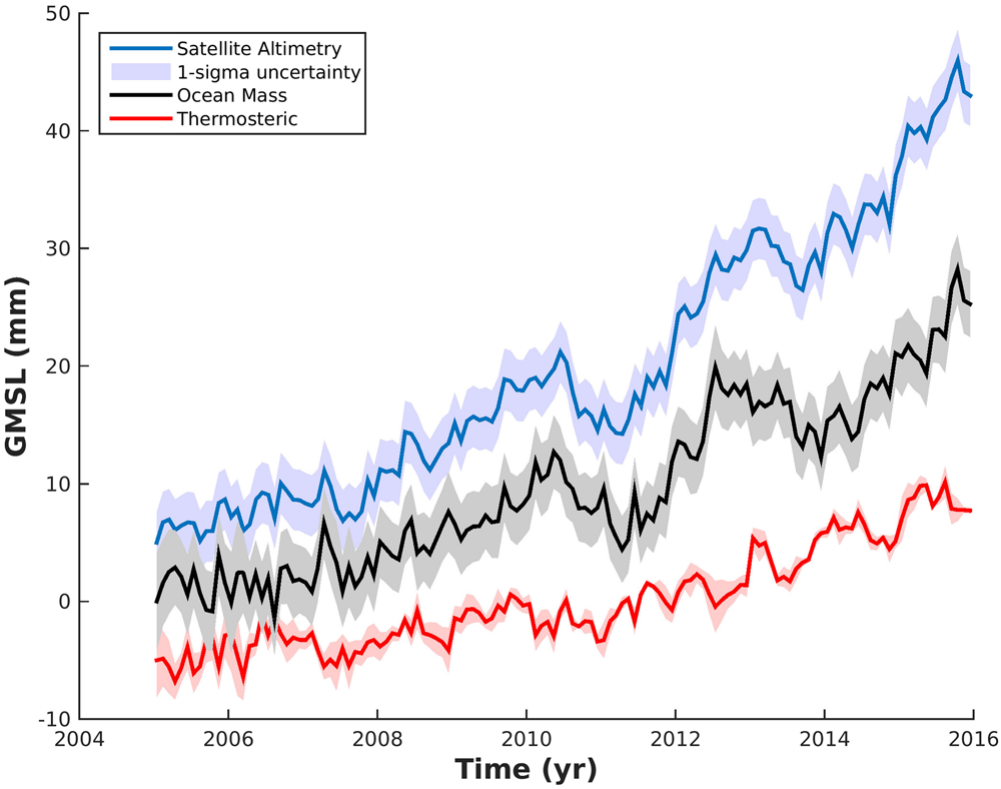
Global mean sea level budget. The net change in sea level observed by satellite altimetry (blue curve) and the thermosteric steric sea level estimated from *in situ* measurements (red). The indirect ocean mass inferred by removing the steric component to the observed sea level time series is shown in black. Seasonal signals have been removed from all curves. Shading denotes 1-σ uncertainty of the respective estimates. Curves are offset for clarity.

Global mean sea level rose at a rate of 3.58 ± 0.25 mm · yr^−1^ (1σ) over 2005–2015 (blue curve in Fig. 1, the error bar comes from an update of ^26^). The quoted trend error always represents the one standard deviation (1σ) unless otherwise stated.

This rise is slightly higher than the entire altimetry period since 1993^8^. This rise slightly decreases to 3.36 ± 0.25 mm · yr^−1^ when computing the global mean sea level change over the Argo-based domain (see method for more details). Global mean sea level shows strong interannual variability around the trend that has been attributed to the fresh water exchanges between oceans and continents during the El-Nino Southern Oscillation events (hereafter ENSO^27,28^) and ocean warming^29^. Full-depth thermosteric sea level shows a linear rise of 1.23 ± 0·18 mm · yr^−1^ (red curve in Fig. 1) corresponding to 37% of the observed sea level rise trend. Our estimate is in line with the recent thermosteric sea level trend estimate of 1.33 ± 0.18 mm · yr^−1^ computed over 2005–2015 with Argo data and hydrographic measurements^30,31^. The upper ocean (above 2000-meter depth) contributes to 1.13 ± 0.02 mm · yr^−1^ estimated mostly on Argo gridded products. (Note that the quoted uncertainty accounts only for the spread based on the Argo gridded products and not for the unsampled regions such as the marginal seas, the high latitude regions and the absence of data under sea ice). The deep ocean (below 2000 meter depth) contributes to 0.10 ± 0.18 mm · yr^−1^ (estimate based on an update of ^32^).

We estimate the ocean mass by removing the net thermosteric component from the observed sea level (black curve in Fig. 1 ^33,34^; see the methods) and by adding 0.1 mm · yr^−1^ to the residual for the elastic response of the Earth^35^. Our ocean mass estimate corroborates the strong contribution of the mass component during the La-Nina event in 2011^28^ with more precipitation over the continents leading to a fall of the global mean sea level. The net ocean mass is increasing with a linear trend of 2.18 ± 0.30 mm · yr^−1^ (assuming the trend errors from satellite altimetry and Argo-based steric trend are not correlated among each other). This value is in line with previous published estimates based on the same approach^7,8^. However, the considered period experienced significant ENSO events, especially the La-Nina in 2011. Therefore, estimating a linear trend over a 11-year time period might not be representative of the long-term change but the interannual variability instead leading to a biased estimate. The ocean mass trend inferred from the sea level budget approach is larger than the continental ice melting budget being 1.67 mm · yr^−1^ over the same time period^11,12^. The latter estimate is smaller by 0.51 mm · yr^−1^. The difference of the net ocean mass inferred from the sea level budget approach and the continental ice melting budget motivates us to reassess the net ocean mass change with an alternative approach. Here, we attempt to provide a novel independent estimate of the ocean mass to further evaluate the net ocean mass uncertainties by assessing the global ocean freshening with global *in situ* data.

### The global ocean freshening

**Figure 2:**
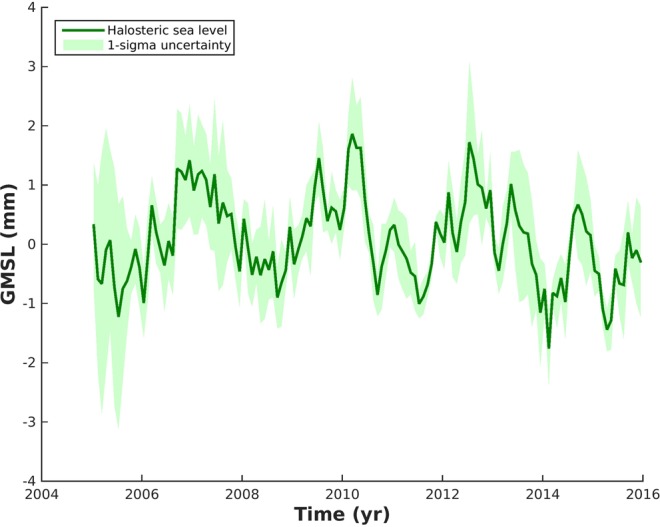
Salinity contribution to sea level. Temporal variability in the halosteric (green curve) sea level estimated from Argo data (0–2000 m). Seasonal signals have been removed from all curves. Shading denotes 1-σ uncertainty of the respective estimates.

For estimating global ocean freshening, we need to estimate the halosteric sea level change. Figure 2 shows the halosteric sea level variations based principally on Argo floats domain (see methods). We find interannual variability for the global mean halosteric sea level change ranging from −2 to 2 mm over 2005–2015 statistically different from zero. This interannual variability is linked to ENSO climate variability over the same time period^36^. We find a halosteric sea level trend of −0.03 ± 0.015 mm · yr^−1^ confirming the fact that halosteric sea level has no significant impact on the global mean seal level rise because the ocean’s total salt content is constant over interannual to decadal timescales and halosteric changes at global scale are due to changes in total freshwater content (ref. ^37^ see Appendix A in^38^ for more details).

The negative halosteric sea level trend is counterintuitive to a freshwater input from continental ice melting that has been largely reported for years^2,7,8^. The negative halosteric sea level trend reflects an increase of the mean ocean salinity for the 0–2000 m depth. However, some areas are not sampled by Argo floats such as the deep ocean, the high latitude regions and the marginal seas^21^. When including the Arctic region, we find a linear trend of 0.0725 ± 0.03 mm · yr^−1^ (formal error from the linear fit), for the 0–2000 m layer, based on the EN4 data only (excluding the regions south of 60°S). This data set merges not only the WOA09 data and Argo floats but also other *in situ* measurements especially to improve data coverage in the Arctic basin with the Arctic Synoptic Basin Wide Oceanography project, the Beaufort Gyre experiment, the North Pole Environmental Observatory (NPEO), the Freshwater Switchyard of the Arctic project, the Nansen and Amundsen Basins Observational System (NABOS) and the Canadian Basin Observational System (CABOS; See^39^ for more discussion).

For the full ocean depth, we need to add the deep ocean contribution below 2000 meter depth. For the deep ocean, we use the hydrographic data between 2000-meter depth and the bottom of the ocean and compute the halosteric sea level change (see the methods). We find a linear trend of 0.007 ± 0.08 mm · yr^−1^ over 1990–2013. The trend error is large due to the lack of *in situ* data. When considering the largest barystatic sea level trend being 2.18 mm · yr^−1^ over 2005–2015 (to be conservative), we find that the full-depth halosteric trend should not exceed 0.0965 mm · yr^−1^ (following the global fresh water budget approach, see method). As the upper halosteric sea level trend is 0.0725 ± 0.03 mm · yr^−1^, the deep ocean halosteric sea level trend cannot exceed 0.017 mm · yr^−1^ in order to not violate the global ocean mass budget. Therefore, we can place a more realistic error bar for the deep halosteric sea level trend that becomes 0.007 ± 0.010 mm · yr^−1^. Even if the trend is not statistically different from zero, we assume this value is representative of the deep halosteric sea level trend over 2005–2015 as the deep ocean circulation is slow and its variability is of a long-term basis. Therefore, our estimate of the full-depth halosteric sea level trend becomes 0.0795 ± 0.032 mm · yr^−1^ over 2005–2015.

The halosteric sea level change accounts for salinity changes due to continental fresh water imports along with floating-ice volume changes from Arctic sea ice and Antarctica sea ice. When corrected for floating-ice volume change the halosteric sea level is directly related to the ocean mass change. Note, here we are asking the question, if estimated changes in ocean salinity due to changes in sea ice where instead from mass input, how much would it change sea level? As we perform a mass budget from observed ocean salinity alone, we need to convert all salinity changes into mass, correcting the sea ice contribution; because we remind the reader that changes in floating sea ice have no effect on actual sea level rise rates.

Significant progress has been made in estimating the floating-ice volume change for the recent years. For Arctic sea-ice, the volume has decreased by 300 ± 100 km^3^ · yr^−1^ ^22^. Recent investigations comparing satellite altimetry and sea ice volume change from PIOMAS shows that the model potentially overestimates the floating sea ice volume by 20%^23^ over 2005–2015. Therefore, we consider the floating sea ice volume change to be 240 ± 100 km^3^ · yr^−1^ as the best estimate. Satellite radar altimeter measurements suggest that the Antarctic ice shelf volume has decreased by 310 ± 37 km^3^ · yr^−1^ for 2003–2012^24^. We assume this trend estimate is representative of the ice shelf volume loss over 2005–2015. Thus, we have a net sea-ice volume decrease of 550 ± 106 km^3^ · yr^−1^ (we assume the trend errors are not correlated among each other). Assuming a mean density of sea-water of 1028 kg · m^−3^ and a mean sea-ice density of 917 kg · m^−3^ ^40^ and considering the Archimedes’ principle (see method), we can convert the net ice volume change into surface height change. We estimate that the linear increase in sea level from the floating sea ice change is 1.36 ± 0.26 mm · yr^−1^. In the process, we neglect the mixing effect (ref. ^14^ for more details see the methods).

**Figure 3:**
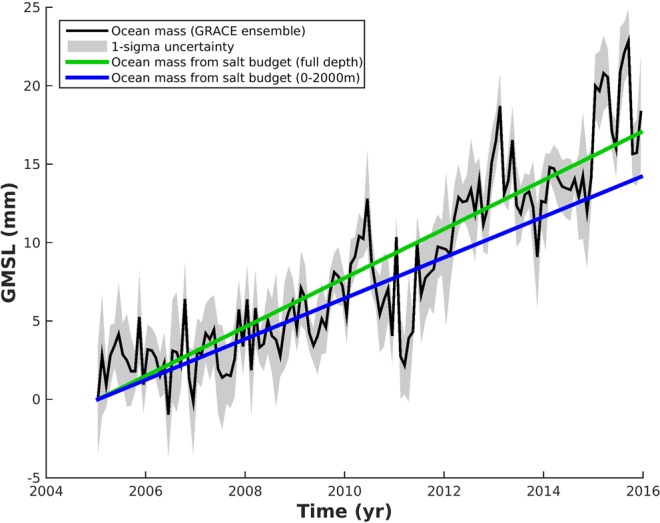
Ocean mass contribution to global mean sea level. Ocean mass change inferred from GRACE data (black curve) and from the global ocean freshening (blue and green curves for the 0–2000 m and full depth, respectively). Seasonal signals have been removed from the GRACE curve. Shading denotes 1-σ uncertainty of the respective estimates. Curves are offset for clarity.

Now we can apply the freshwater budget approach to the full-depth halosteric sea level trend. We multiply the halosteric trend by the Munk’s factor (which is 36.7) and we remove the sea level trend due to sea ice melt. Therefore, for the 0–2000 m layer, we find an ocean mass trend of 1.30 ± 1.13 mm · yr^−1^ (blue curve in Fig. 3) and for the full-depth, we find a net ocean mass trend of 1.55 ± 1.20 mm · yr^−1^ (green curve in Fig. 3). We assume the trend errors from halosteric sea level trend and floating sea-ice melting are not correlated among each other.

The full-depth ocean mass trend estimate is in line with recently published ocean mass solution derived from GRACE data (ref. ^10^; black curve in Fig. 3). The ocean mass inferred by GRACE, in Fig. 3, presents some interannual variability that is linked to the ENSO events^27,28^. The ocean mass deduced from the global ocean freshening is within the uncertainty of the GRACE-based ocean mass. We find a linear increase of 1.60 ± 0.16 mm · yr^−1^ for the ocean mass inferred by GRACE over 2005–2015. Note however that these new estimates are smaller than the previous ocean mass estimates recently published that are based on the sea level budget approach^2,7,8^.

## Discussion and Conclusions

Evaluating the freshening of the ocean provides a unique and indirect estimation of the net ocean mass change. Based on the unprecedented amount of salinity data from Argo floats along with historical *in situ* measurements from oceanographic campaigns, we find an increase of the net ocean mass of 1.55 ± 1.20 mm · yr^−1^ using a full ocean depth freshening trend over 2005–2015.

Interestingly, our results are in line with new ocean mass trends recently published of 1.60 ± 0.27 mm · yr^−1^ based on an ensemble of GRACE data (10, see methods) and of 1.67 mm · yr^−1^ based on continental ice melting budget reassess from satellite observations and energy input-output model over January 2005–December 2015^11,12^. This agreement demonstrates the usefulness of investigating the global salt budget to assess the net ocean mass trend over the ocean. However our results are smaller by 0.63–0.88 mm · yr^−1^ than the ocean mass trend of 2.18 ± 0.30 mm · yr^−1^ deduced from the sea level budget approach (observed mean sea level minus the thermosteric component). These different estimates are not statistically different from each other denoting remaining large uncertainties. More investigations are needed to lower down these large uncertainties.

Our global ocean freshening analysis is based on some important hypothesis that might present some limitations. Wang *et al*.^36^ already highlight large spread among the Argo gridded products while assessing the halosteric sea level trend since 2005. They find a negative trend of −0.05 mm · yr^−^ not in line with recent freshwater import from continents such as observed ice sheet mass loss and mountain glaciers melting. They speculate some possible reasons for this questioning negative trend. Some possible explanations might be: (i) the freshwater import affects the high latitude regions (not sampled by Argo floats) and needs more than one decade to be detected in the tropical and mid-latitude oceans, (ii) evaporation minus precipitation might contribute on decadal time scales in addition to continental fresh water imports (from continental ice and land water variation), (iii) the interannual variability might dominate the decadal trend over a short time period, (iv) the deep ocean below 2000 meters might contribute to explain the difference, and (v) the marginal seas are not sufficiently sampled by Argo floats over the past 11 years. We have addressed some of these limitations by including *in situ* data from the deep ocean and from the Arctic region. However, more investigations are needed to fully address the remaining limitations.

Continuous records of *in situ* temperature and salinity based on Argo floats and other *in situ* measurements are essential to refine the decadal trend estimates for the thermosteric and halosteric sea level variations and to lower down the associated uncertainties for the steric sea level change and subsequently the ocean heat content and freshwater budget to close the recent sea level budget.

In addition, the Argo network is not global and some regions are not well sampled. Continuous hydrographic missions and new observing systems are needed to continuously sample the temperature and salinity changes for the deep ocean (where we highlight large uncertainty in our analysis), the high latitude regions and the marginal seas. Some developments are underway such as the deployment of the recent deep Argo floats (that reach the 4000 to 6000 meters of the ocean ground depending on the probe) is a step forward in reducing the errors^41^. These new floats will continuously monitor the deep ocean evolution that is needed to narrow the uncertainties in the ocean heat content change, the freshwater budget and the sea level budget at global and regional scales. Hence, in the near future, the sampling issues discussed above will be addressed for the deep ocean based on new “deep-Argo” floats. While our analysis offers a major improvement, in terms of observational sampling, over previous studies^14,15^ the poorly sampled parts of the ocean remain a limitation.

Major improvements have been made in estimating the Arctic sea-ice and Antarctic ice shelf volume changes for the recent years. Such estimates are essential to evaluate with good accuracy the net ocean mass change from the global ocean freshening approach as the floating sea-ice equivalent sea level change is of the same order of magnitude than the land ice freshwater inputs. Continuous efforts are needed to refine these estimates and therefore reduce the trend errors to better ascertain the global ocean freshening and therefore the barystatic sea level changes.

Last, we have assumed that each observing system is independent and that errors are uncorrelated over time scales longer than one month. If this assumption is not true, then, the error estimate quoted in our analyses might be underestimated.

Estimating as accurate as possible sea level variations and its causes are of great interest not only to constrain both the Earth’s water cycle and energy budget, but also to ascertain the climate models used to predict future sea level evolutions.

Our results provide an entirely independent constraint on the global ocean mass trend budget. As GRACE data over the oceans are sensitive to the combination of geocenter motions and GIA, our results provide bounds for the magnitude of the required corrections for the trend estimate. Estimating with good accuracy both the geocenter variations and Glacial Isostatic Adjustment over the oceans is an important challenge for the scientific community for the coming years.

## Methods

### Sea level equation

Based on the hydrostatic equation, sea level anomalies corrected from barometric changes can be partitioned into the barystatic sea level changes (i.e., net ocean mass changes – Δh_Barystatic_) and thermosteric sea level changes (density variations) within the water column (Δh_Thermosteric_) following the sea level equation^33^:

$$\Delta {H}_{SeaLevel}=\Delta {H}_{Barystatic}+\Delta {H}_{Thermosteric}$$

where

$$\Delta {H}_{Thermosteric}=-\,{\int }_{-H}^{0}\,\frac{\rho (z)-{\rho }_{ref}(z)}{{\rho }_{0}}dz$$

Here ρ(z) is the sea water density, ρ_ref_ is the referenced sea water density (T = 0 °C and S = 35 psu), ρ_0_ is a reference density, H depth of the ocean, and z vertical coordinate of the water column. The anomalies (Δ) are defined relative to associated time mean.

### Global ocean salt budget and mass budget

The global mean salinity of the ocean has decreased slightly over 1954–1997^13^. This global ocean freshening is linked to imports of continental freshwater to the oceans (from ice sheets mass loss and mountain glaciers melting) and floating sea level volume shrinks. Therefore, estimating the global ocean freshening provides a unique and independent estimate of the barystatic sea level change when correcting for the floating sea ice volume change (in term of sea level).

The net ocean mass is estimating following the methodology described by Munk^14^.

$$\Delta {H}_{Barystatic}+\Delta {H}_{SeaIce}=\frac{\rho }{\Delta \rho }\Delta {H}_{Halosteric}=36.7\Delta {H}_{Halosteric}$$

where ΔH_Barystatic_ is the sea level change due to continental fresh water inputs along with floating sea ice change, ρ is the mean sea level density (1028 kg · m^−3^) and Δρ is the difference between the mean sea level density and the fresh water density (28 kg · m^−3^). Therefore, ΔH_SeaIce_ is the sea level change associated with floating sea ice change. Floating sea ice change does not affect sea level change because of the Archimedes’ principle. However, it changes the salinity balance and needs to be accounted for while assessing the global freshwater budget of the oceans.

The net halosteric sea level change is estimated as followed:

$$\Delta {H}_{Halosteric}=\Delta {H}_{0-2000m}+\Delta {H}_{2000m-bottom}$$

where ΔH_0–2000m_ represents the halosteric sea level change from *in situ* data (Argo floats plus the hydrographic data) for the upper 2000 meters. ΔH_2000m-Bottom_ is the halosteric sea level contribution from the hydrographic data for the deep ocean part (following the methodology from^32^).

The equivalent sea level change due to floating sea-ice can be easily deduced following the Archimedes’ principle. The weight conservation equation can be written as follow:

$${W}_{SeaIce}={W}_{Ocean}$$

where W_SeaIce_ is the floating sea ice weight and W_Ocean_ is the equivalent displacement of sea water. The equation can be developed as:

$$g{M}_{Ice}=g{M}_{Ocean}$$

where g is the acceleration of gravity and M_ice_ is the floating sea ice mass and M_ocean_ is the corresponding sea water mass.

Therefore, the equation can be written as:

$${\rho }_{SeaIce}{V}_{SeaIce}={\rho }_{Ocean}{V}_{Ocean}$$

$${\rho }_{SeaIce}{V}_{SeaIce}={\rho }_{Ocean}{V}_{Ocean}={\rho }_{Ocean}{S}_{Ocean}{H}_{SeaIce}$$

and finally,

$${H}_{SeaIce}=\frac{{\rho }_{SeaIce}{V}_{SeaIce}}{{\rho }_{Ocean}{S}_{Ocean}}$$

where ρ_Sea Ice_ is the mean sea-ice density equal to 917 kg · m^−3^, ρ_Ocean_ is the mean density of sea water of 1028 kg · m^−3^, S_Ocean_ is the surface of the ocean being 360 × 10^6^ km^2^ and V_Sea Ice_ is the floating sea-ice volume change. We find a sea level change associated with floating sea ice change of H_SeaIce_ = 1.36 ± 0.26 mm · yr^−1^.

Therefore, we can assess the sea level trend due to the net ocean mass as:

$$\Delta {H}_{Barystatic}=36.7\Delta {H}_{Halosteric}-\Delta {H}_{SeaIce}$$

where ΔH_Barystatic_ is the net global ocean mass due to continental freshwater inputs.

### Sea level data

Sea level has been regularly measured by satellite altimetry since 1992 with the launch of TOPEX/Poseidon followed by Jason-1 and -launched in 2001 and 2008, respectively. This family of satellites provides a near-global coverage (+/−66° of latitude) of the oceans every ten days. We use four gridded products: (i) Colorado University (CU released 5, http://sealvel.colorado.edu), (ii) Goddard Flight Space Center^42^, (iii) the Climate Change Initiative (CCI) sea level data (ftp.esa-sealevel-cci.org/Products/SeaLevel-ECV/)^43^ and (iv) AVISO (AVISO website https://www.aviso.altimetry.fr/en/data/products/ocean-indicators-products/mean-sea-level.html). Instrumental and geophysical corrections have been applied to the datasets. In addition, a correction of −0.3 mm · yr^−1^ has been applied to account for the effect of the Glacial Isostatic Adjustement (GIA^44^) to the CCI product. The CCI product includes data from ERS-1/2 and Envisat along with the aforementioned satellite data and is based on a new approach that reduces orbit errors, wet/dry atmospheric correction errors, reduction of instrumental drifts and bias, inter-calibration biases and between satellite, and an improved reference of the mean sea surface (for more detail, see^26^).

### Steric sea level data

Steric sea level is the sum of the upper ocean steric estimated from Argo and deep ocean steric estimated from repeat hydrography.

### Steric sea level (0–2000): Argo gridded products

We use in this study gridded temperature and salinity data that are obtained from four separate groups: (i) Scripps Institution of Oceanography (hereafter SIO, updated from^45^), (ii) EN4^39^ and (iii) JAMSTEC^46^ and (iv) ISAS15 from LOPS laboratory (updated from^47^). These datasets can be downloaded at www.argo.ucsd.edu/Gridded_fields.html. SIO dataset uses Argo float data only whereas the other groups combine not only Argo floats, but also other *in situ* measurements (for example, expendable bathythermograph -XBT-, Conductivity-Temperature-Depth -CTD- and mooring data). Argo-based temperature and salinity data have been passed through real time and delayed time quality control checks (see the Argo quality control manual for more detail).

Steric sea-level time series are computed by using temperature and salinity data from each dataset. We consider the Thermodynamic Equation of Sea Water (http://www.teos-10.org) as the equation of state. For more detail on the computation, see^34^. We have removed a monthly climatology defined as the time-mean over the respective time periods for each calendar month as we focus our analyses on interannual to decadal changes.

### Steric >2000: Deep ocean data

The deep (below 2000 m) steric contribution to the global mean sea level rise rate is evaluated using high quality, full-depth, ship-based CTD data collected either through the World Ocean Circulation Experiment (WOCE) hydrographic program or the Global Ocean Ship-Based Hydrographic Investigations Program (GO-SHIP). Data was collected from the sea surface to within 10 m of the bottom nominally every 55 km along all transects and maintained the highest quality of salinity, temperature and pressure measurements with accuracy of 0.002, 0.001 °C and 10 dbar or better, respectively. Salinity was calculated from the CTD and calibrated to bottle samples standardized with International Association of the Physical Science of the Oceans (IAPSO) standard seawater using the 1978 practical salinity scale (PSS-78).

The halosteric deep ocean trend was found following the method used to calculate the global deep thermosteric component in^48^ (hereafter P&J 2013) but done globally. The sections data grid the global ocean was screened and gridded following P&J 2013. At each vertical and horizontal grid point along the section, we calculate a linear rate of change in salinity with time (dS/dt). Sections are divided into 32 deep ocean basins defined by topography and bottom water properties again following P&J 2013, and basin means and standard deviations are calculated at each depth along isobars using all available data within the basin. Each basin’s halosteric expansion and error below 2000 m is calculated using the basin mean and standard deviation with a locally derived halosteric contraction coefficient, integrated from the bottom to 2000 m. The global mean deep halosteric sea level trend below 2000 m is the sum of the volume change in the 32 basins divided by the surface area of the ocean. Here, basins with no data are assumed to have no change in salinity as is any region of the basin deeper than the deepest sampled measurement.

The error around the mean is again evaluated following P&J 2013. The basin’s standard deviation at each depth for each basin is converted into a halosteric expansion by multiplying by halosteric contraction coefficient and integrating vertically. The basin STD is converted into a standard error (SE) using the basin’s degrees of freedom (DOF), calculated by the length of all sections across the basin divided by a horizontal length scale of 163 km following P&J 2013. The standard error of the 32 basins are added in quadrature and divided by the surface area of the ocean.

### GRACE data

The direct estimation of ocean mass contribution to sea level change is based on an ensemble of GRACE data. We consider in this study the GRACE LEGOS V1 solutions^10^. The dataset consists of an ensemble of 1500 solutions that considers variation on 6 different processing parameters namely the processing centers (CSR -Center for Space Research-, GFZ—GeoforschungsZentrum-, JPL—Jet Propulsion Laboratory-, GRGS—Groupe de Recherche de Geodesie Spatiale-, and TUG—Graz University of Technology), the geocenter motion^49–53^ and C(2, 0) coefficient^54,55^ corrections, the filtering^56,57^, the leakage correction over a 300-km-wide zone off the coastlines based on comparison with observation-based ocean mass estimates (2 ocean estimates based on^58,59^) and the GIA correction^60–62^.

### Data processing

As we consider many gridded products in the paper with different spatial domains, we have decided to interpolate the *in situ* gridded products (EN4, JAMSTEC and ISAS15) to the SIO spatial domain. For the sea level budget analysis, we have estimated the global mean sea level trend over the entire domain and over the SIO domain. From 2005 to 2015, we find a trend difference of 0.22 mm · yr^−1^ considering the CCI gridded product. Therefore, we have removed the latter value to the observed global mean sea level time series in the study.

All estimates in the present study are anomalies with respect to their time-mean. The curves are offset for clarity. Each curve has an envelope around the mean estimate denoting the one standard deviation computed with all the considered datasets. To estimate uncertainty in the trend, we perform a weighted least-squares fit at a monthly basis. The weights are chosen to equal the reciprocal of the square of the measurement accuracy for each month^63^. The degrees of freedom are equal to 130 as we fit a linear trend over 2005–2015 at a monthly basis (i.e., 132 observations).

## Data Availability

Sea level data are freely available at http://sealvel.colorado.edu, ftp.esa-sealevel-cci.org/Products/SeaLevel-ECV/ and, https://www.aviso.altimetry.fr/en/data/products/ocean-indicators-products/mean-sea-level.html and ftp.esa-sealevel-cci.org/Products/SeaLevel-ECV/. The Argo gridded products are freely available at http://www.argo.ucsd.edu/Gridded_fields.html. The CTD data for WOCE and GO-SHIP international programs are freely available at https://cchdo.ucsd.edu/. The ensemble of GRACE are can be downloaded at http://www.legos.obs-mip.fr/en/share/soa/cgi/getarc/v0.0a/index.pl.cgi?contexte=SOA&donnees=gravimetrie&produit=grace_legos.
